# Single-cell RNA-seq and *in vitro* study reveal *Fusobacterium nucleatum* impairs β-cell identity in type 2 diabetes via the NF-κB–CDKN1C axis

**DOI:** 10.1186/s12967-026-07981-x

**Published:** 2026-03-09

**Authors:** Ziyi Wei, Tianqi Xu, Xiufeng Gu, Qi He, Qiang Feng, Meihui Li

**Affiliations:** Department of Human Microbiome, School and Hospital of Stomatology, Cheeloo College of Medicine, Shandong University and Shandong Key Laboratory of Oral Tissue Regeneration and Shandong Engineering Research Center of Dental Materials and Oral Tissue Regeneration and Shandong Provincial Clinical Research Center for Oral Diseases, No.44-1 Wenhua Road West, Jinan, Shandong 250012 China

**Keywords:** *Fusobacterium nucleatum*, β-cell identity, CDKN1C, NF-κB pathway, Type 2 diabetes

## Abstract

**Background:**

The pathogenesis of type 2 diabetes is characterized by insulin resistance and a progressive decline in β-cell function. A key driver of this dysfunction is the loss of β-cell identity, which reduces functional β-cell mass and leads to inadequate insulin secretion. Periodontal pathogens have been implicated in promoting insulin resistance; however, their role in the transformation of β-cell identity remains poorly understood. This study aims to investigate the impact of periodontal pathogen *Fusobacterium nucleatum* (*F. nucleatum*) on β-cell identity maintenance and the underlying molecular mechanisms.

**Method:**

Single-cell RNA sequencing (scRNA-seq) data from human pancreatic islets of nondiabetic (ND), prediabetic (Pre-T2D), and type 2 diabetes (T2D) donors were analyzed to assess changes in β-cell proportion, differentiation trajectory, and associated molecular pathways. The Single-cell Analysis of Host-Microbiome Interactions (SAHMI) method was used to detect *F. nucleatum* sequences in pancreatic islets. Pearson correlation analysis identified key genes associated with the action of *F. nucleatum*, followed by in vitro validation using a co-culture model of *F. nucleatum* and MIN6 cells to elucidate the underlying mechanisms.

**Results:**

scRNA-seq analysis revealed a reduced proportion of β-cells and decreased expression of key β-cell identity-maintenance genes in the T2D group. The expression levels of transdifferentiation markers and β-cell disallowed genes were elevated, alongside a trend toward α-cell transdifferentiation. The NF-κB signaling pathway was significantly activated in the T2D group, accompanied by a significant increase in the SPP1 inflammatory signal, while the WNT pathway was markedly diminished. Integrated Pearson correlation and in vitro analyses identified the cell cycle regulator CDKN1C as a central mediator through which *F. nucleatum* promotes β-cell identity loss. Mechanistically, *F. nucleatum* activated the NF-κB pathway, leading to downregulation of CDKN1C expression and thereby promoting loss of β-cell identity, which played an important role in the progression of diabetes associated with periodontitis.

**Conclusion:**

This study demonstrates that β-cells in T2D primarily undergo transdifferentiation towards α-cells, and the periodontal pathogen *F. nucleatum* promotes β-cell identity loss via NF-κB-mediated downregulation of CDKN1C.

**Supplementary information:**

The online version contains supplementary material available at 10.1186/s12967-026-07981-x.

## Backgroud

Type 2 diabetes (T2D) is a globally widespread metabolic disease characterized primarily by chronic hyperglycemia resulting from defects in insulin secretion and/or insulin resistance [1, 2]. In the progression of T2D, sustained metabolic stress leads to progressive impairment of β-cell function [3, 4]. A key pathological feature of T2D is the progressive loss of functional β-cells in both number and secretory capacity, closely associated with β-cell identity loss, marked by a diminished mature β-cell phenotype, significant downregulation of key transcription factors, and impaired insulin synthesis and secretion [5–9]. Additionally, markers of endocrine progenitor cells or other islet cell types (e.g., α-cells, δ-cells) are aberrantly upregulated, further disrupting β-cell homeostasis [10, 11]. Elucidating the molecular mechanisms of β-cell identity loss in T2D may provide critical insights for improving long-term patient outcomes.

Periodontitis is not merely a localized oral disease but also a significant risk factor for T2D [12]. Studies have shown that Gram-negative bacteria or their virulence factors within dental plaque can disseminate to distant organs, leading to metabolic endotoxemia and insulin resistance [13–15]. For example, lipopolysaccharide (LPS) from *Porphyromonas gingivalis* (*P. gingivalis)* can disrupt systemic immune responses in mice, and its gingipains promote insulin resistance by degrading insulin receptors on multiple cell types [16, 17]. Moreover, infections caused by pathogens such as *Fusobacterium nucleatum* (*F. nucleatum*) and *Prevotella intermedia* (*P. intermedia*) have been linked to worsened metabolic dysfunction and arterial hypertension in experimental diabetes [18]. Prior research has largely focused on *P. gingivalis* and its effects on glycemic control and insulin resistance; recent findings demonstrate that *F. nucleatum* colonization accelerates the onset of diabetes in mice [19]. Therefore, further investigation into the impact of *F. nucleatum* on β-cell function and identity maintenance may provide critical insights into the mechanisms underlying periodontitis-associated exacerbation of T2D.

This study investigated whether and how the periodontal pathogen *F. nucleatum* contributes to T2D progression by promoting β-cell identity loss. We first performed bioinformatics analyses using a human T2D pancreatic islet scRNA-seq dataset. Through quantification of cell proportions, we characterized dynamic alterations in the β-cell population during T2D progression. Pseudotemporal trajectory analysis was employed to trace the lineage trajectories and evolutionary patterns of β-cell transdifferentiation associated with disease advancement. Next, bacterial annotation analysis confirmed the presence of *F*. *nucleatum* in the pancreatic islet. Multiple experimental and analytical approaches were integrated to elucidate the key molecular pathways and regulatory mechanisms underlying pathogen-mediated β-cell identity loss. Collectively, this study provides direct evidence supporting the functional involvement of oral microbiota in the pathogenesis of T2D.

## Materials and methods

### Data acquisition and sequence alignment

In order to investigate the unique biological characteristics of human islet cells in different states, we analyzed the scRNA-seq data of human islets from nondiabetic (ND), prediabetes (Pre-T2D), and type 2 diabetes patients (T2D). The scRNA-seq data were obtained from the Gene Expression Omnibus (GEO) database (GSE221156). The scRNA-seq data were aligned with the human genome (GRCh38) using cellranger (v7.1.0) [20], and the count matrix was generated.

### Data quality control and cell clustering

scRNA-seq data were processed using Seurat (v5.0.1) [21]. During quality control, cells meeting any of the following criteria were excluded: fewer than 500 unique molecular identifiers (UMIs), fewer than 200 genes detected, or mitochondrial gene content exceeding 20%. Potential doublets were identified and removed using DoubletFinder (v2.0.3) [22]. Sample integration was performed with Harmony (v1.2.0) to correct for batch effects [23]. Dimensionality reduction and visualization were carried out using Uniform Manifold Approximation and Projection (UMAP) [24], and cell clusters were identified based on established marker genes from the literature. Differential gene expression across clusters was assessed using the FindAllMarkers function. Cluster-specific highly expressed genes and enriched pathways for the top 150 marker genes were visualized using the ClusterGVis package (v0.1.1). Pathway enrichment significance results were retained based on a multiple-testing corrected Storey *q*-value of less than 0.05.

### AUCell scoring and slingshot pseudotemporal analysis

The proportions of all cell types within the ND, Pre-T2D, and T2D groups were calculated relative to the total number of cells in each group. Additionally, the proportions of β-, α-, δ-, and PP cells within each sample were summarized at the sample level. The expression of key transcription factors, dedifferentiation markers, β-cell-disallowed genes, and β-cell maturity markers in β-cell subpopulations across the three groups was evaluated using AUCell scoring (1.24.0) [25]. A comprehensive AUCell score was computed by integrating the expression levels of representing β-cell identity maintenance and non-β-cell markers. At the sample level, we have assessed the effects of potential covariates using a linear model and adjusted for sex, BMI, chemistry, and race, then compared the adjusted scores across groups.

Pseudotemporal analysis was performed for the endocrine cell clusters in the three groups using Slingshot trajectory analysis (v2.10.0) [26], which enabled a comparison of cell type transition trajectories among the groups. We evaluated the trajectory weights of β-cells along each lineage and measured the proportion of high-weight β-cells (Weight > 0.8) within each trajectory at the sample level to determine which trajectory β-cells are more inclined towards. At the sample level, we have assessed the effects of covariates on pseudotime values using linear models and adjusted for age, sex, BMI, and race before comparing pseudotime values across the three groups. Statistical significance between groups was assessed using the Wilcoxon test.

### Differential gene enrichment and cell-cell communication analysis in β-cells across ND and T2D groups

Differentially expressed genes between the ND and T2D groups were identified using the FindMarkers function with the MAST method, while adjusting for age, sex, BMI, chemistry, and race. The filtering criteria for differentially expressed genes in β-cells between the ND and T2D groups were set as: adjusted *p*-value < 0.05 and fold change > 1.2. Functional enrichment analysis of differentially expressed genes in β-cells between the ND and T2D groups was performed using the clusterProfiler package (v4.10.0). The significantly enriched pathways (multiple-testing corrected Storey *q*-value < 0.05) for different gene sets were visualized using dot plots. Additionally, gene set enrichment analysis (GSEA) was conducted based on gene expression changes between the ND and T2D groups to compare pathway activation patterns, and the significantly enriched pathways (multiple-testing corrected Storey *q*-value < 0.05) were shown in a bar plot.

Cell-cell communication analysis was carried out with CellChat (v1.6.1) [26], and differences in communication networks between the ND and T2D groups were systematically compared. Signaling clusters were identified based on functional similarity, and signaling networks with larger (or smaller) differences were discerned according to the Euclidean distance in a shared two-dimensional space. By comparing the outgoing (or incoming) signaling patterns between the two groups, signaling pathways exhibiting distinct communication patterns were identified. Furthermore, communication probabilities mediated by ligand-receptor pairs between β-cells and other cell types were compared, leading to the identification of signaling pairs that were either up-regulated (enhanced) or down-regulated (diminished) in the T2D group.

### *F. nucleatum* annotation and correlation analysis with genes

Sequences not aligned to the human reference genome were extracted using Samtools (v1.17) [27], and microbial information in the single-cell data was subsequently obtained via the Single-cell Analysis of Host-Microbiome Interactions method (SAHMI) [28]. The unaligned FASTQ files were processed with Kraken2 (v2.1.3) [29] against the Standard database to extract microbial sequences. After barcode-level signal denoising (barcode k-mer correlation tests on taxonomy IDs detected on > 3 barcodes and with >1 k-mer) and sample-level signal denoising (using default parameters), combining k-mer correlation tests and filtering significant values and species resolution to obtain the real microbial sequence. Microbial information was then integrated with host RNA data, and UMAP visualization was used to display the expression distribution of *F. nucleatum*. Differential gene expression analysis was performed between β-cells with and without *F. nucleatum* expression, retaining results meeting the criteria of adjusted *p*-value < 0.25, *p*-value < 0.05, and fold change > 1.2. Functional enrichment analysis was subsequently conducted on the resulting gene set, with results showing *p*-value < 0.01 and *q*-value < 0.25 being retained for visualization in the bubble plot.

Partial correlation analysis was conducted to assess the association between the abundance of *F. nucleatum* and differentially expressed genes in β-cells from both ND and T2D groups while controlling for covariates BMI. Genes that are significantly correlated with *F. nucleatum* (*p* < 0.05) and expressed in more than 50% of cells in the T2D group were retained and visualized using the lollipop plot. Genes showing negative correlations were selected for KEGG and GO enrichment analyses, and the significantly enriched pathways (multiple-testing corrected Storey *q*-value < 0.05) were shown in a bar plot. Based on the screened gene CDKN1C, its expression levels were compared between the ND and T2D groups, and Pearson correlations were calculated between CDKN1C and genes associated with non-β-cell markers as well as β-cell maturity markers. An adjusted *p*-value of less than 0.05 was considered statistically significant.

### Verification dataset

The GSE164416 transcriptomic dataset from GEO was used for validation, sharing the same sample type and a similar size to our study, with 18 ND and 39 T2D samples. Human gene sequences were aligned to GRCh38 using bowtie (2–2.5.1), and unaligned sequences were analyzed with Kraken2 (v2.1.3) to identify microbial sequences. *F. nucleatum* annotation was extracted, and abnormal samples were removed. The proportion of samples annotated to *F. nucleatum* in the ND and T2D groups was calculated. β-cell identity loss scores were computed using single sample GSEA (ssGSEA) based on related genes representing β-cell identity maintenance and non-β-cell markers, and regression analysis assessed the relationships between β-cell identity loss scores, *F. nucleatum* abundance, and CDKN1C expression.

### Bacteria and cell lines

*F.nucleatum* ATCC 25,586 strain was provided by the Shandong Provincial Key Laboratory of Oral Tissue Regeneration (Jinan, China). Bacterial cultures were grown in Brain Heart Infusion (BHI; BD Bioscience, USA) complete medium supplemented with vitamin K₁ (Haibo Biological) and hemin (Haibo Biological). Cultivation was carried out anaerobically at 37 °C for 3 days, after which bacteria were harvested by centrifugation at 2655 × g for 5 minutes.

The mouse pancreatic β-cell line MIN6 was purchased from BeNa Culture Collection (BNCC, China). Cells were maintained in high-glucose Dulbecco’s Modified Eagle Medium (DMEM) supplemented with 10% fetal bovine serum (FBS; Gibco, USA) and 1% penicillin-streptomycin (Gibco, USA), with subculturing performed every 2–3 days.

### Apoptosis assay

MIN6 cells were co-cultured with *F.nucleatum* at varying MOIs (0, 50, 100, 200) for 24 hours. After PBS washes, cells were resuspended in 1× Annexin V Binding Solution and stained with Annexin V-FITC and PI (Beyotime, Shanghai, China) for 15 minutes in the dark. Apoptosis was quantified by flow cytometry within 1 hour.

### MOI and bacterial viability verification

The multiplicity of infection (MOI) and bacterial viability during co-culture were validated by colony-forming unit (CFU) assays. Bacterial suspensions with an OD value of 1 or bacterial-cell co-culture systems were serially diluted and plated onto BHI agar plates. Bacterial viability during co-culture was further assessed by CFU enumeration at different time points.

### Real-time quantitative polymerase chain reaction (RT-qPCR)

Total RNA was extracted from cells using Trizol reagent (CWBIO, Beijing, China). cDNA synthesis was performed with the *Evo M-MLV* Reverse Transcription Premix Kit (Accurate Biotechnology, Hunan, China). RT-qPCR was conducted using the SYBR Green *Pro Taq* HS Premix qPCR Kit (Accurate Biotechnology, Hunan, China), and relative mRNA expression levels of target genes were analyzed using the 2^(−ΔΔCt) method, normalized to β-actin. The primer sequences used in this process are detailed in Table S13.

### Glucose-stimulated insulin secretion (GSIS)

Cells were pre-treated with *F.nucleatum* for 24 hours, washed with PBS, and incubated in glucose-free KRBH buffer for 1 hour. Insulin secretion was induced by treatment with 2.8 mM or 16.7 mM glucose for 1 hour. Supernatants were collected, and insulin levels were measured using an ELISA Kit (Zcibio, Shanghai, China).

### Cell counting kit-8 (CCK-8) assay

Cell viability was assessed using the CCK-8 assay (C0037, Beyotime) following the manufacturer’s instructions. Cells were seeded into 96-well plates at a density of 1 × 10^4^ cells per well. After adherence, cells were treated with *F. nucleatum* for the indicated durations, followed by the addition of 20 μL of CCK-8 solution to each well. After incubation at 37 °C for 1–4 h, the absorbance was measured at 450 nm. Cell viability was expressed as a percentage relative to untreated control cells.

### In vitro gene silencing and plasmid transfection

MIN6 cells were seeded into 6-well plates and incubated in a culture medium with 10% FBS overnight, then transfected with siRNA using Rfect V2 siRNA Transfection Reagent (BIOG, Changzhou, China) in complete medium. The following sequences of siRNA are listed in Table S13.

MIN6 cells were cultured in 6-well plates and transfected with plasmids using Lipofectamine 3000 (Invitrogen, USA) in Opti-MEM medium (Genom, China). Plasmid schematics are shown in the Supplementary Material.

### Western blot analysis

MIN6 cells were lysed with fresh RIPA buffer containing protease and phosphatase inhibitors. Protein concentrations were quantified using a BCA Protein Assay Kit (EpiZyme, China). Equal amounts of protein were separated by SDS-PAGE gel (EpiZyme, China) and transferred onto PVDF membranes (Millipore, Billerica, USA). Membranes were blocked with 5% non-fat milk, incubated overnight at 4 °C with primary antibodies (Table S13), followed by HRP-conjugated secondary antibodies. Protein bands were visualized using an Amersham Imager 680 (GE, USA) and analyzed with ImageJ, which used β-actin as the internal control.

### Chromatin immunoprecipitation (CHIP)

Chromatin immunoprecipitation assays were conducted using the Chromatin Immunoprecipitation Kit (BersinBio) following the manufacturer’s protocol. In short, cells were cross-linked with 1% formaldehyde, followed by quenching with glycine. The chromatin was sheared by sonication to fragments ranging from 300 to 1000 bp. The soluble chromatin supernatant was diluted and incubated overnight at 4 °C with a specific antibody (shown in Table S13) or control IgG, with a portion set aside as the Input control. Antibody-bound complexes were captured using protein A/G-beads and then reversed at 65 °C in elution buffer for 6 hours, after which DNA was isolated. Enrichment of specific genomic regions was quantified by RT-qPCR using SYBR Green and sequence-specific primers (shown in Table S13).

### Statistical analysis

Each cytology experiment was performed three times in biological replicates, and the results were presented as the mean ± standard deviation (SD). For comparisons between two groups, an unpaired two-tailed Student’s t-test was used. For multi-group comparisons involving a single independent variable, a one-way ANOVA was performed, followed by Dunnett’s post hoc test (each treatment vs. control) or Tukey’s test (all pairwise comparisons), as appropriate. For experiments involving two independent variables, two-way ANOVA was used, followed by Tukey’s multiple-comparisons test as appropriate. Analyses were conducted using GraphPad Prism 9.5, and *p* < 0.05 was considered statistically significant.

## Results

### T2D exacerbated β-cell identity loss and transdifferentiation toward α-cells

**Fig. 1:**
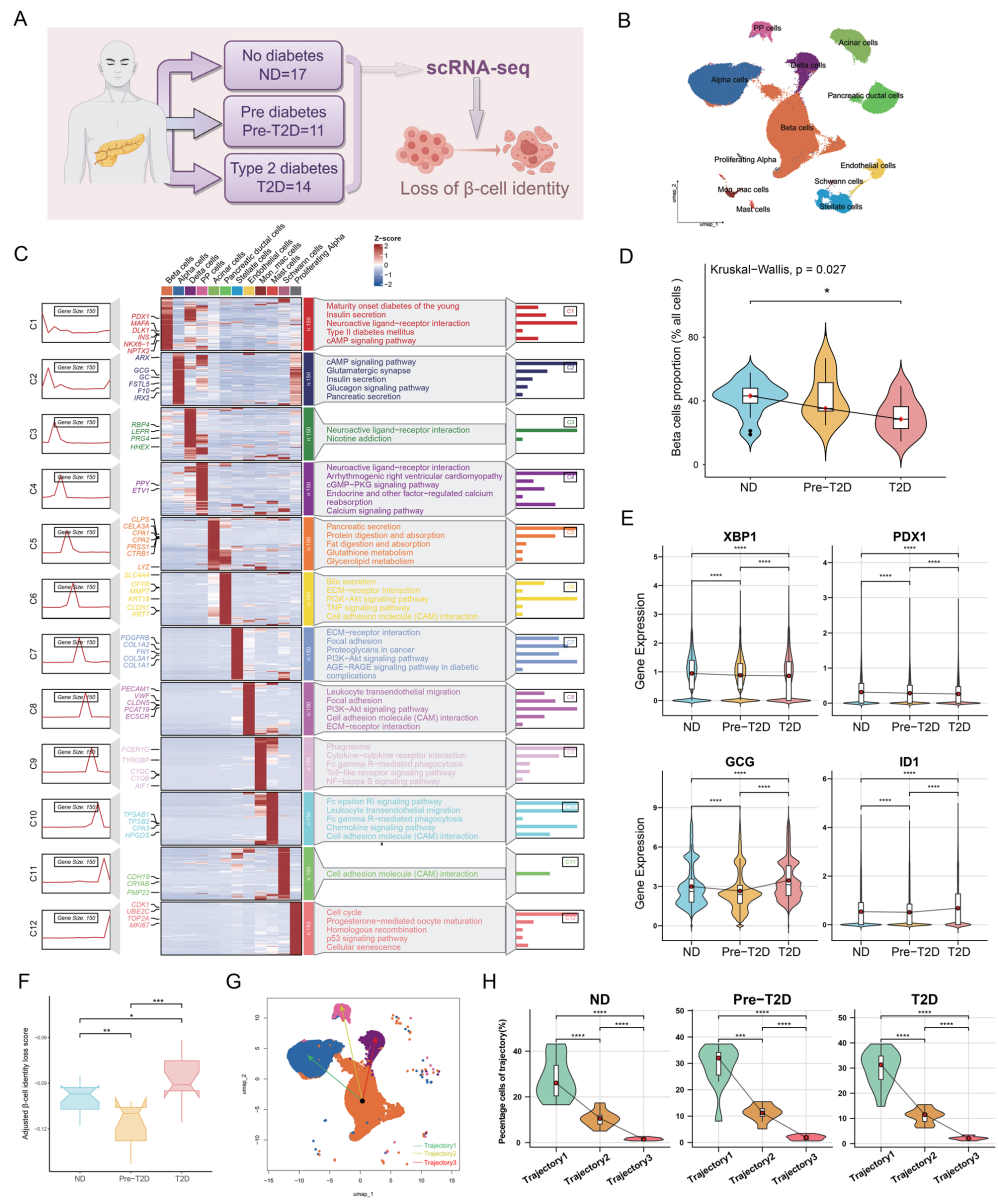
Single-cell profiling reveals β-cell identity loss in T2D. (**A**) The schematic depicts the grouping information. (**B**) UMAP showing the 12 main clusters in different colors. (**C**) The top 150 highly abundant genes and their corresponding enriched KEGG pathways in each islet cell cluster. Marked genes are used for the annotation of cell clustering. (**D**) β-cell composition in ND, Pre-T2D, and T2D groups. Red dots indicate the median values. Significance was assessed by Kruskal-Wallis (*p* = 0.027) and pairwise Wilcoxon tests (*p ≤ 0.05). (**E**) XBP1, PDX1, GCG, and ID1 gene expression within β-cells across ND, Pre-T2D, and T2D groups. Red dots indicate the mean values (Wilcoxon test, ****p ≤ 0.0001). (**F**) Box plot showing the β-cell identity loss scores among the ND, Pre-T2D, and T2D groups at sample level (Wilcoxon test, *p ≤ 0.05, ** *p* < 0.01, and ***p < 0.001). (**G**) Slingshot trajectory analysis of β-, α-, δ-, and PP cells in ND, Pre-T2D, and T2D groups. Different colors of lines correspond to distinct trajectories. (**H**) Proportion of high-weight β cells in three trajectories across groups. Red dots indicate the median values (Wilcoxon test, ****p ≤ 0.0001)

To investigate alterations in pancreatic islet cell composition during the progression of T2D, we analyzed scRNA-seq data from 42 human islet samples obtained from the GEO dataset (GSE221156), including 17 samples from ND donors, 11 from Pre-T2D donors, and 14 from T2D donors (Fig. 1A) and their clinical information (Table S1). After removing doublets (Table S2) and filtering out low-expression genes, a total of 262,667 high-quality cells were retained, comprising 97,120 cells in the ND group, 85,932 in the Pre-T2D group, and 79,615 in the T2D group. Cell clustering was performed based on global gene expression profiles, and cell types were annotated using 61 established marker genes, leading to the identification of 12 distinct cell populations (Fig. S1A and 1B). KEGG pathways enrichment analysis of the top 150 marker genes highlighted key signaling pathways such as insulin secretion and cAMP signaling (Fig. 1C and Table S3). GO analysis indicated that biological processes were predominantly associated with regulation of insulin secretion and response to glucose (Fig. S1B and Table S4), while molecular functions included calcium channel activity and adenylate cyclase activity (Fig. S1C and Table S5). These findings underscore the cellular heterogeneity and functional complexity of the pancreatic islet.

Subsequently, we analyzed the shifts in the proportions of pancreatic cell types in the Pre-T2D and T2D groups. UMAP analysis revealed distinct clustering patterns for the twelve cell types across the three groups (Fig. S2A). In the T2D group, the β-cell proportion decreased from 43% to 32% (Fig. S2B), whereas α-cells increased from 26% to 31%, and PP cells from 2.3% to 3.3% (Fig. S2B). Other cells, such as pancreatic duct cells, increased from 7% to 11%, and pancreatic stellate cells increased from 3.8% to 6.7% (Fig. S2B). Notably, compared to the ND group, the β-cell proportion was significantly reduced in the T2D group at the sample levels (Fig. 1D). The α-cells showed a gradually increasing trend, while the δ- and PP cells showed a decreasing trend (Fig. S2C). These findings demonstrated an imbalance in the composition of islet endocrine cells and an expansion of fibrotic cell types under T2D conditions.

We further examined the expression of genes associated with β-cell identity maintenance across the ND, Pre-T2D, and T2D groups [30, 31]. PDX1, a core transcription factor essential for β-cell function and identity maintenance [32], was significantly downregulated in both the Pre-T2D and T2D groups (Fig. 1E). Similarly, XBP1, a gene critical for sustaining normal insulin biosynthesis in β-cells [33], was markedly downregulated (Fig. 1E). Other key transcription factors involved in maintaining mature β-cell identity, including PAX6, MAFA, INSM1, FOXA2, and HNF4A [34–36], also showed a downregulated trend in T2D (Fig. S2D). Notably, the expression of the GCG and ID1 in β-cells was significantly elevated in the T2D group compared to the ND group (Fig. 1E), and genes including LDHA, ID2, ARX, ALDH1A3, and NANOG exhibited upward trends (Fig. S2E). These results indicated that during T2D progression, β-cells undergo transcriptional dysregulation characterized by loss of identity-associated factors.

We applied AUCell scoring to evaluate the extent of β-cell identity loss in the ND, Pre-T2D, and T2D groups. The results revealed a significant reduction in β-cell identity maintenance (Fig. S2F), accompanied by a marked upregulation of non-β-cell markers in the T2D group (Fig. S2G). Combined assessment of these two gene sets (representing β-cell identity maintenance and non-β-cell markers) demonstrated a substantial increase in β-cell identity loss in the T2D group (Fig. 1F). To further validate our observations, we analyzed an additional human islet transcriptome dataset (GSE164416, including 18 ND samples and 39 T2D samples) by applying the gene sets defined in Fig. S2D and S2E. The analysis similarly revealed an increasing trend in the loss of β-cell identity within the T2D group (Fig. S2H and S2I). Slingshot trajectory analysis identified three transition paths originating from β-cells (Fig. 1G). We evaluated β-cell trajectory weights along each lineage and quantified, at the sample level, the proportion of high-weight β-cells within each trajectory. A higher proportion of β-cells was distributed along the trajectory toward α-cell identity (Fig. 1H). Based on the average weights calculated across all β-cells, trajectory 2 and trajectory 3 contained a large number of β-cells along the trunk of the pseudotime trajectory (Fig. S2J). We compared pseudotime values of high-weight β-cells across trajectories and found that, along the α-cells-directed trajectory, β-cells from the T2D group exhibited a less stable transcriptional state (Fig. S2K). Collectively, these findings indicated that under T2D conditions, β-cells experience functional deterioration and are predisposed to transdifferentiate toward α-cells.

### T2D enhanced inflammatory signals and impaired identity maintenance pathways in β-cells

**Fig. 2:**
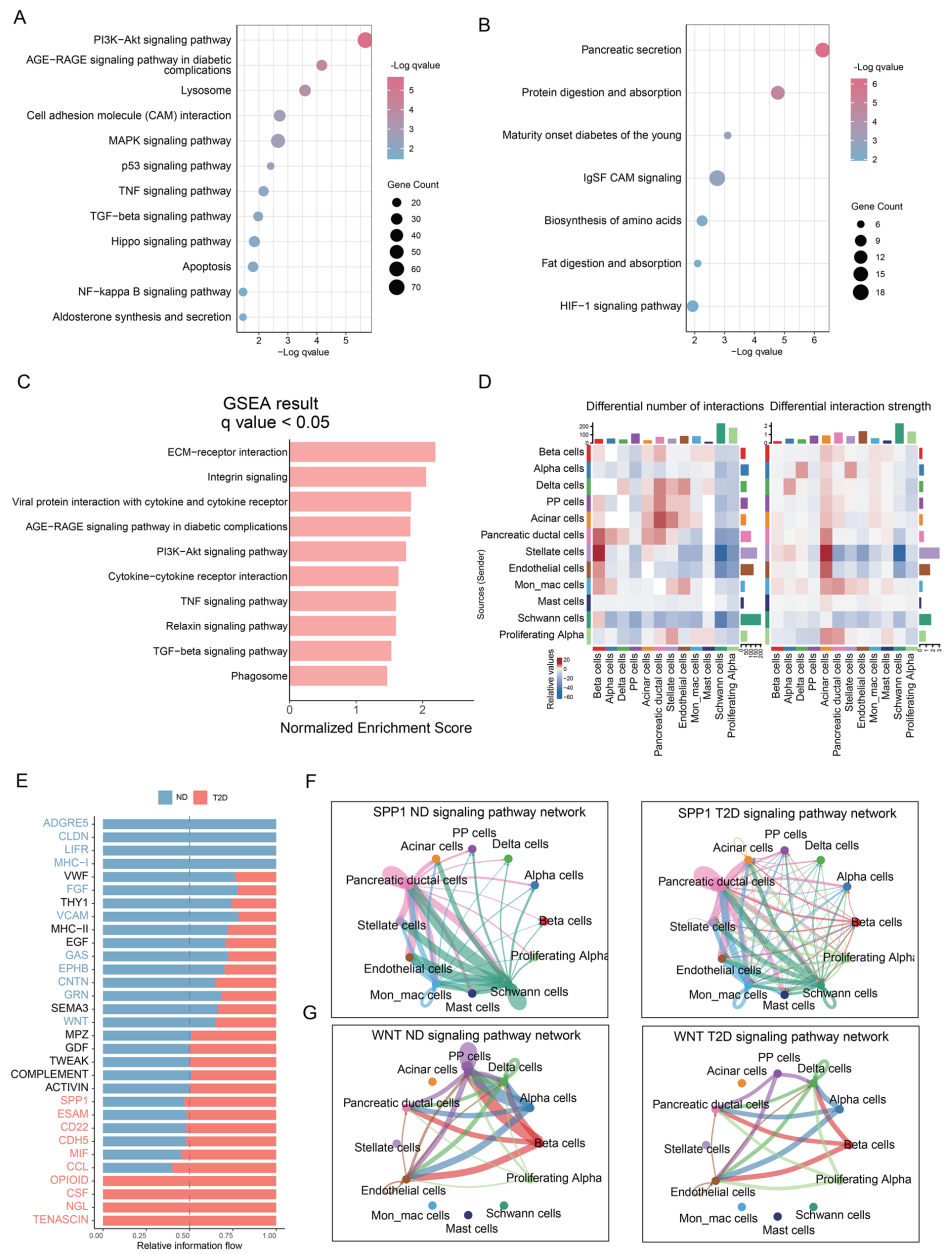
Alterations in signaling pathways and cell communication in diabetic β-cells. (**A**) The up-regulated genes in β-cells and their enriched pathways in the T2D group compared with the ND group (*q* < 0.05). (**B**) The down-regulated genes and their enriched significant pathways of β-cells in the T2D group (*q* < 0.05). (**C**) Barplot showing the GSEA results of β-cells in T2D versus ND groups (*q* < 0.05). (**D**) The heatmap shows the differential number of interactions and the differential interaction strength of cell communication between the ND and T2D groups. Red color: T2D group ＞ND group; blue color: T2D group＜ND group. (**E**) The stacked bar chart shows the top 15 signaling pathways enriched in the ND group and the top 15 signaling pathways in the T2D group, respectively. (**F**), (**G**) The network showing the intercellular communication of SPP1 (**F**) and WNT (**G**) Signaling pathway in ND and T2D groups. The thicker the line, the stronger the signal. The color of the connection line is determined by the cell type that is the source signal

To investigate the signaling pathways associated with β-cell identity loss in T2D, we compared transcriptomic profiles and pathway activities in β-cells between ND and T2D groups. In the T2D group, upregulated genes were predominantly enriched in TNF, PI3K-Akt, Hippo, and NF-κB signaling pathways (Fig. 2A and Table S6). Conversely, genes significantly downregulated in the T2D group were primarily enriched in Pancreatic secretion, Protein digestion and absorption, and HIF-1 signaling pathways (Fig. 2B and Table S7). GSEA confirmed significant activation of pathways in the T2D group (Table S8), including TNF (Fig. S3A and 2C), NF-κB (Fig. S3B), and PI3K-Akt signaling pathway (Figs. 2C and S3C). These results indicated that under T2D conditions, the function of pancreatic cells is in a state of decline.

To investigate whether the functional alterations of β-cells in T2D were correlated with the cell-cell communication, we used CellChat to quantify the intercellular communication among 12 cell clusters. The results showed that in the T2D group, both the number and strength of communication signals from β-cells to pancreatic duct cells, stellate cells, endothelial cells, and monocytes were elevated, whereas signals between β-cells and Schwann cells or proliferating α-cells were reduced (Fig. 2D). Next, we performed a functional similarity analysis on the signaling pathways commonly present in both the ND and T2D groups, classifying them into four distinct functional clusters (Fig. S3D), and identified differential signal networks by assessing their Euclidean distances. Notably, the SPP1 signaling pathway exhibited the most pronounced difference in activity between the ND and T2D groups (Fig. S3E). The information flow of the signaling pathway revealed that the SPP1 pathway was enriched in the T2D group, while the WNT pathway was enriched in the ND group. (Fig. 2E). Specifically, SPP1 signaling output was significantly upregulated in β-cells in the T2D group (Figs. 2F and S3F), and other cell types, such as pancreatic duct cells, stellate cells, and monocytes, also exhibited increased signaling activity (Figs. 2F and S3F). Next, we analyzed the ligand-receptor interactions associated with the SPP1 signaling pathway across β-cells and other cell types. The results showed that several SPP1 receptors, including CD44, ITGAV+ITGB5, and ITGAV+ITGB1, were significantly upregulated in α-cells, duct cells, stellate cells, and monocytes in the T2D group (Fig. S3G). We observed that the WNT pathway, which is essential for islet homeostasis and function integrity [37], exhibited markedly reduced communication between β-cells and PP cells in the T2D group (Fig. 2G). The expression of WNT4 receptor complexes, including FZD6+LRP6, FZD6+LRP5, FZD3+LRP6, and FZD3+LRP5, was significantly decreased in PP cells (Fig. S3G). Subsequently, we further analyzed the expression of SPP1 and WNT4 in β-cells between the ND and T2D groups. The results revealed that SPP1 expression was significantly upregulated in the T2D group (Fig. S3H), whereas WNT4 expression was significantly downregulated (Fig. S3I). These results indicated that T2D increases inflammatory signals in the pancreas and impairs critical supporting signals required for maintaining β-cell identity.

### *F. nucleatum* correlated with β-cell identity loss and CDKN1C expression

**Fig. 3:**
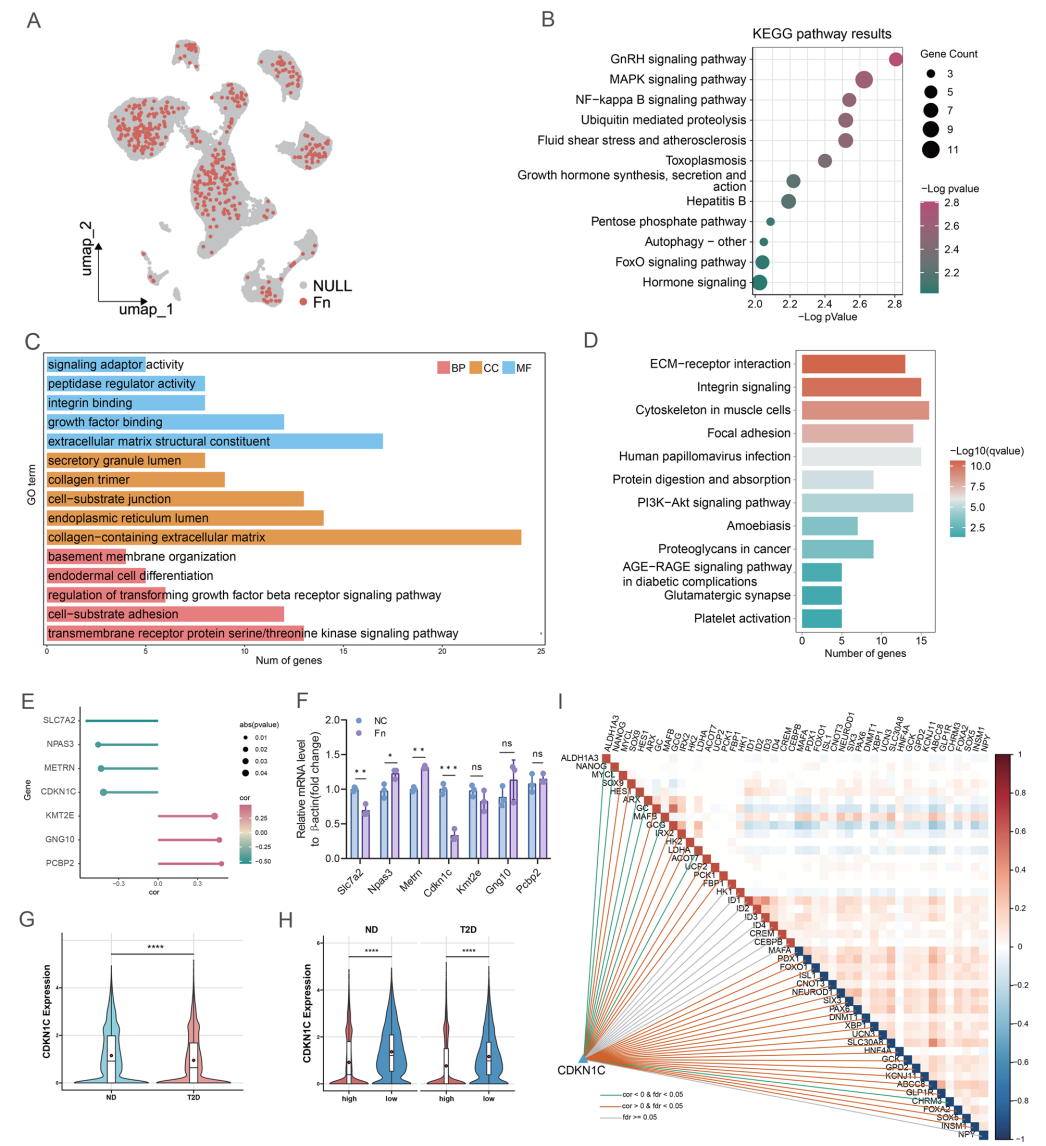
The identification of key genes correlates with *F. nucleatum* and β-cell identity loss. (**A**) UMAP showing cells with *F. nucleatum* RNA sequence annotated by SAHMI. (**B**) KEGG enrichment analysis of DEGs between *F. nucleatum-* and non- *F. nucleatum-* annotated β-cells in the T2D group (*p*-value < 0.01 and *q*-value < 0.25). (**C**) Barplot showing the enrichment results of genes negatively correlated with *F. nucleatum* in β-cells from both ND and T2D groups (*q*-value < 0.05). The color is determined by the three domains: BP, CC, and MF. (**D**) KEGG enrichment results of genes negatively correlated with *F. nucleatum* in β-cells of ND and T2D groups (*q*-value < 0.05). (**E**) Genes associated with *F. nucleatum* in β-cells (*p* < 0.05 and the expression percentage in T2D group > 50%). (**F**) RT-qPCR analysis of changes in *F. nucleatum* -associated genes expression. (**G**) Expression of CDKN1C in β-cells of ND and T2D groups. Red dots indicate the mean values (Wilcoxon test, ****p ≤ 0.0001). (**H**) Expression of CDKN1C stratified by the extent of β-cell identity loss in the ND and T2D groups. Red dots indicate the mean values (Wilcoxon test, ****p ≤ 0.0001). (**I**) The Pearson correlations between CDKN1C and genes involved in β-cell identity. The green line: cor < 0 and FDR < 0.05, the orange line: cor > 0 and FDR < 0.05, the gray line: FDR ≥ 0.05. On the heatmap diagonal, red rectangles denote genes with high expression supporting β-cell identity loss, blue ones denote genes highly expressed in β-cell maturation

To investigate whether β-cell identity loss in T2D is associated with *F. nucleatum*, we annotated bacterial sequences within pancreatic islets using SAHMI. UMAP analysis demonstrated the presence of *F. nucleatum* in islet cells (Fig. 3A). In the transcriptome dataset GSE164416, the presence of *F. nucleatum* was also confirmed. The annotation rate was approximately 70% across all samples. (Fig S4A). KEGG pathway analysis of DEGs in β-cells with or without *F. nucleatum* revealed significant enrichment in pathways related to the NF-κB, FOXO, and Hormone signaling pathways (Fig. 3B and Table S9), indicating that *F. nucleatum* may be associated with impaired β-cell function. We next examined the correlation between *F. nucleatum* and the DEGs of β-cells to identify key transcriptional alterations associated with β-cell identity loss. Compared to the ND group, most DEGs in the T2D group exhibited a negative correlation with *F. nucleatum* abundance (Table S10). GO enrichment analysis indicated that these DEGs were predominantly involved in the peptidase regulator activity and endodermal cell differentiation (Fig. 3C and Table S11). KEGG pathway analysis revealed that *F. nucleatum* correlated genes were enriched in the PI3K-Akt and Protein digestion and absorption (Fig. 3D), which are implicated in insulin secretion and cell differentiation [38–40]. These results suggested that *F. nucleatum* may substantially influence β-cell function.

Subsequently, we identified seven genes that were both highly abundant in the T2D group and correlated with *F. nucleatum* (Fig. 3E and Table S10). These findings were validated by RT-qPCR analysis in MIN6 cells, which revealed a marked downregulation of CDKN1C expression upon *F. nucleatum* stimulation (Fig. 3F). CDKN1C is consistently downregulated in β-cells undergoing aberrant differentiation [41, 42]. We investigated the role of CDKN1C in β-cell identity maintenance under *F. nucleatum* exposure. The expression of CDKN1C was significantly reduced in the T2D group (Fig. 3G), and its abundance showed a markedly negative correlation with the degree of β-cell identity loss (Fig. 3H). Pearson correlation analysis revealed that CDKN1C expression was positively correlated with β-cell identity markers, such as MAFA, PDX1, and FOXO1 (Fig. 3I and Table S12). In contrast, markers of endocrine progenitor cells such as NANOG and HES1, along with β-cell disallowed genes (ALDH1A3, LDHA) and the α-cell marker ARX, were negatively associated with CDKN1C (Fig. 3I and Table S12). In the transcriptome dataset GSE164416, we further verified the relationship between *F. nucleatum* and CDKN1C and β-cell identity loss. Both calculation methods (based on the genes in Fig. S2E or Figs. S2D and S2E) showed that the abundance of *F. nucleatum* was positively correlated with the degree of β-cell identity loss (Figs. S4B and S4C), and the expression level of CDKN1C was negatively correlated with the degree of identity loss (Fig. S4D). These findings suggested that *F. nucleatum* is correlated with β-cell identity loss and CDKN1C expression.

### *F. nucleatum* promoted the loss of β-cell identity

**Fig. 4:**
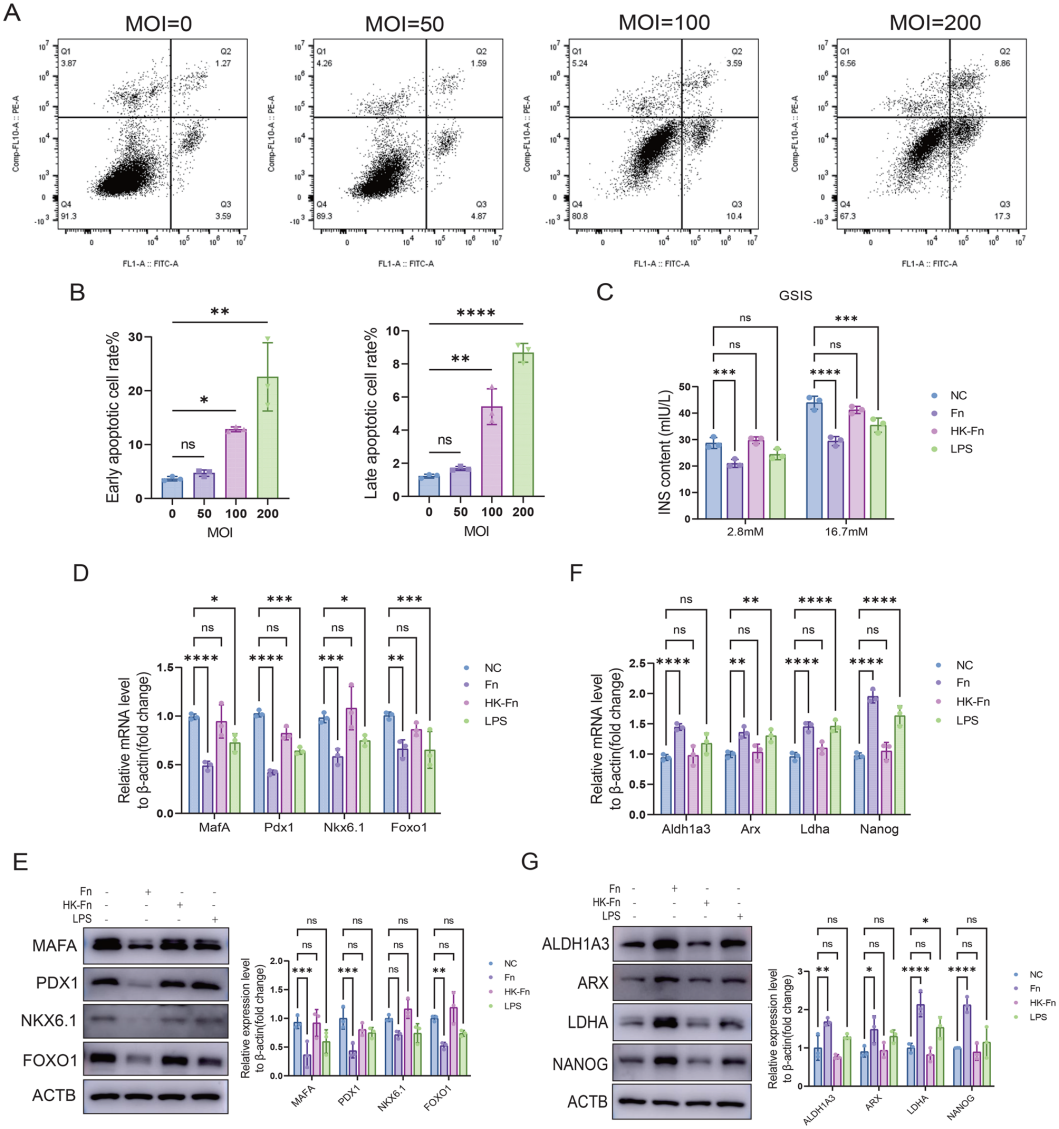
*F. nucleatum* promotes β-cell identity loss. (**A**), (**B**) Flow cytometry analysis of cell apoptosis under different MOI of *F. nucleatum*. (**C**) GSIS assay in MIN6 cells treated with *F. nucleatum*, HK-Fn, and LPS for 24 h, subsequently treated with 2.8 mM or 16.7 mM glucose. Insulin levels were determined by ELISA. (**D**) RT-qPCR analysis and Western blot (**E**) of MAFA, PDX1, NKX6.1, and FOXO1 in MIN6 cells treated with *F. nucleatum*, HK-Fn, and LPS for 24 h. (**F**) RT-qPCR analysis and Western blot (**G**) of ALDH1A3, ARX, LDHA, and NANOG by *F. nucleatum*, HK-Fn, and LPS infection for 24 h. Data were shown as mean ± SD. One-way ANOVA (**B**) and two-way ANOVA (**C-G**) Were used to examine the statistical significance between groups. *p < 0.05, **p < 0.01, and ***p < 0.001

To validate whether *F. nucleatum* could promote β-cell identity loss, we conducted in vitro experiments using MIN6 cells. We performed Annexin V/PI double staining followed by flow cytometry to determine the optimal infection concentration. The results showed that when the multiplicity of infection (MOI) exceeded 50, both early and late apoptotic MIN6 cells were significantly increased (Figs. 4A and 4B). CFU assay confirmed 39 viable *F. nucleatum* at MOI 50, persisting throughout MIN6 co-culture (Figs. S5A and S5B). RT-qPCR showed significant downregulation of β-cell function genes by 16 h, stabilizing at 24 h (Fig. S5C). GSIS and CCK8 assays indicated 24 h as the optimal time for consistent insulin secretion reduction without compromising viability (Fig. S5D and S5E). Thus, MOI 50 (39 CFU) and 24 h co-culture were chosen for all subsequent *F. nucleatum* experiments.

To dissect whether the pathogenicity of *F. nucleatum* is dependent on its viability or bacterial components, we included four groups: a negative control, live *F. nucleatum*, heat-killed *F. nucleatum* (HK-*Fn*), and LPS. GSIS was used to assess β-cell function. The results demonstrated that insulin secretion from β-cells was markedly reduced following *F. nucleatum* exposure across various glucose concentrations (Fig. 4C). In contrast, HK-*Fn* elicited no significant effect. Although LPS treatment also reduced insulin secretion, the extent of this reduction was less than that induced by live *F. nucleatum* infection (Fig. 4C). Further analysis by RT-qPCR and Western blot revealed that *F. nucleatum* significantly downregulated both mRNA and protein expression levels of key mature β-cell identity markers (MAFA, PDX1, FOXO1, and NKX6.1) (Figs. 4D and 4E). HK-*Fn* exerted no significant effect, and the impact of LPS on these genes was also weaker than that of *F. nucleatum* (Figs. 4D and 4E). Moreover, *F. nucleatum* strongly upregulated the expression of endocrine progenitor cell markers (NANOG), α-cell marker (ARX), and β-cell disallowed genes (ALDH1A3, LDHA) (Figs. 4F and 4G). Similarly, HK-*Fn* produced no observable effect, while LPS, which increased the mRNA levels of these markers, did not significantly alter their protein expression (Figs. 4F and 4G). These findings indicated that *F. nucleatum* stimulation can induce significant β-cell dysfunction and loss of cellular identity, and this pathogenic effect depends on the bacterial activity. Therefore, in the subsequent studies, we mainly focused on the active form of *F. nucleatum* to investigate its pathogenic effects.

### *F. nucleatum* promoted β-cell identity loss via CDKN1C downregulation through the NF-κB pathway

**Fig. 5:**
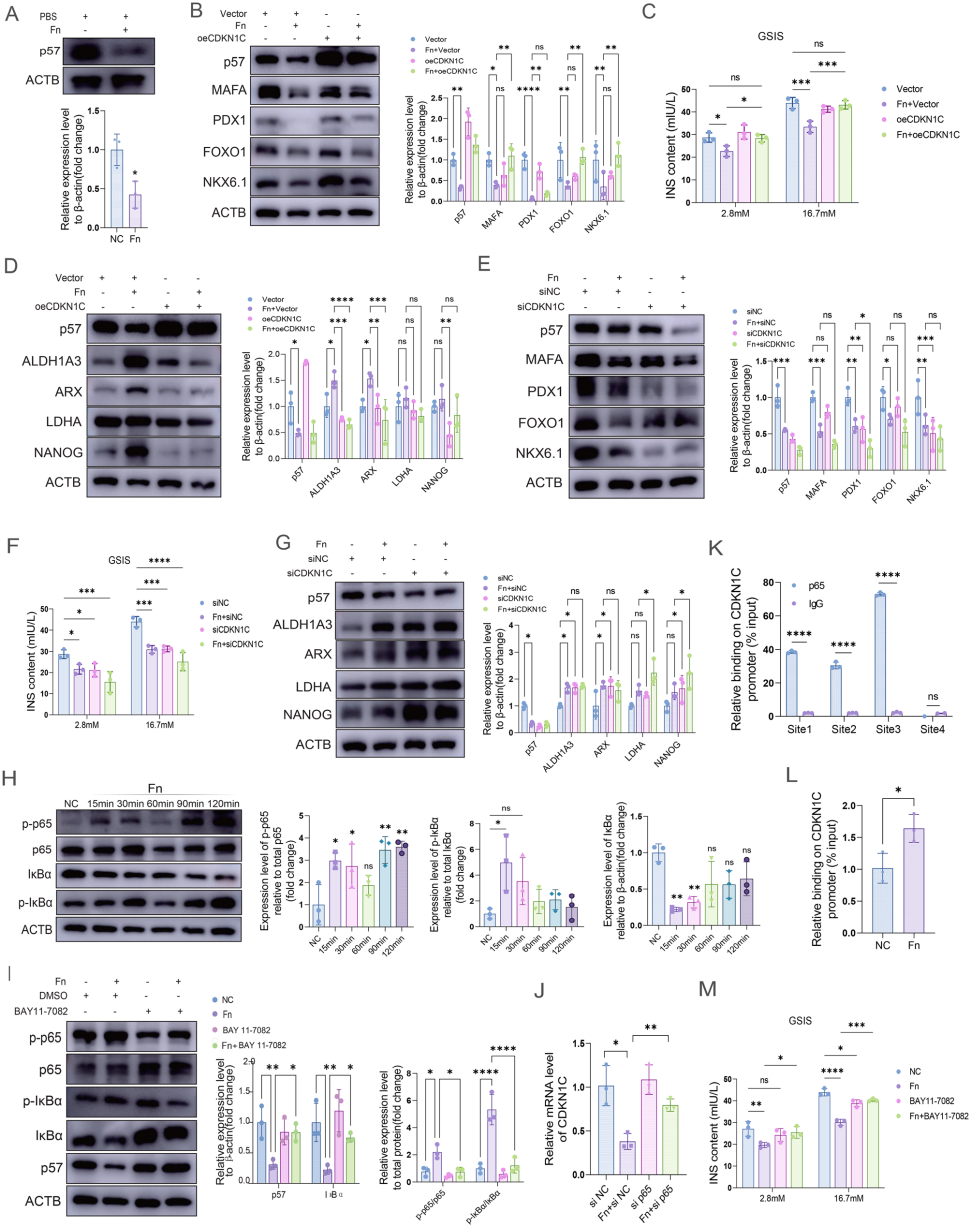
*F. nucleatum* promotes β-cell identity loss via the NF-κB pathway and CDKN1C downregulation. (**A**) Western blot of p57 after *F. nucleatum* infection for 24 h. (**B**) Western blot showing the impact of oeCDKN1C and *F. nucleatum* infection on MAFA, PDX1, FOXO1, and NKX6.1. (**C**) GSIS assay in MIN6 cells treated with *F. nucleatum* and oeCDKN1C. Insulin levels were determined by ELISA. (**D**) Western blot showing the impact of oeCDKN1C and *F. nucleatum* infection on ALDH1A3, ARX, LDHA, and NANOG. (**E**) Western blot of MAFA, PDX1, FOXO1, and NKX6.1 in MIN6 cells preincubated with siCDKN1C and treated with *F. nucleatum*. (**F**) GSIS assay in MIN6 cells treated with *F. nucleatum* and siCDKN1C. Insulin levels were determined by ELISA. (**G**) Western blot of ALDH1A3, ARX, LDHA, and NANOG in MIN6 cells preincubated with siCDKN1C and treated with *F. nucleatum*. (**H**) Western blot of p-p65, p65, p-IκBα, IκBα after *F. nucleatum* infection 15 min, 30 min, 60 min, 90 min, and 120 min. (**I**) Western blot of p-p65, p65, p-IκBα, IκBα, and p57 by *F. nucleatum* infection and BAY11-7082 treatment. (**J**) RT-qPCR analysis of the mRNA levels of CDKN1C treated with *F. nucleatum* and sip65. (**K**) ChIP-qPCR was performed with an antibody for p65 and an antibody for IgG as the negative control. (**L**) ChIP-qPCR was performed in MIN6 cells with *F. nucleatum* exposure. (**M**) GSIS assay in MIN6 cells preincubated with BAY11-7082 and treated with *F. nucleatum*. Insulin levels were determined by ELISA. Data were shown as mean ± SD. Student’s t test (**A, K** and **L**), one-way ANOVA (**H**), and two-way ANOVA (**B-G, I, J, M**) were used to examine the statistical significance between groups, *p < 0.05, **p < 0.01, and ***p < 0.001

We further investigated the role of CDKN1C in β-cell identity loss mediated by *F. nucleatum*. Western blot analysis showed that *F. nucleatum* exposure significantly reduced CDKN1C protein expression (Fig. 5A). To investigate whether *F. nucleatum* induces β-cell identity loss through CDKN1C, we performed transient overexpression and knockdown of CDKN1C in MIN6 cells using plasmid vectors and siRNA, respectively (Fig. S5F and S5G). Functional validation revealed that CDKN1C overexpression effectively reversed the downregulation of mature β-cell identity markers (MAFA, FOXO1, NKX6.1) (Fig. 5B) and insulin secretion level induced by *F. nucleatum* (Fig. 5C), and concurrently suppressed the upregulation of β-cell identity loss indicators, such as the disallowed gene ALDH1A3 and LDHA, the α-cell marker ARX, and the endocrine progenitor marker NANOG (Fig. 5D). Conversely, CDKN1C knockdown further exacerbated the inhibitory effects of *F. nucleatum* on key β-cell transcription factors (MAFA, PDX1, FOXO1, NKX6.1) (Fig. 5E) and insulin secretion (Fig. 5F), while significantly increasing the expression of LDHA and NANOG. (Fig. 5G). These results suggested that CDKN1C plays an important role in *F. nucleatum*-induced β-cell identity loss.

As shown in Figs. 2A and 3B, the NF-κB signaling pathway was enriched in β-cells from the T2D group and related to the dedifferentiation of β-cells. Therefore, we examined the impact of *F. nucleatum* on the NF-κB pathway and its relationship with CDKN1C expression and insulin secretion level. The results indicated that 15 minutes after *F. nucleatum* infection, total IκBα protein levels were markedly decreased, whereas phosphorylation of p65 and IκBα was significantly increased (Fig. 5H), indicating activation of the NF-κB signaling pathway in β-cells stimulated with *F. nucleatum*. To determine whether the expression level of CDKN1C depends on NF-κB pathway activation, we pretreated cells with BAY11-7082 to block pathway activity. Notably, BAY11-7082 treatment not only suppressed p65 phosphorylation and prevented IκBα degradation but also substantially restored CDKN1C expression (Fig. 5I). To further investigate whether the NF-κB pathway regulates CDKN1C transcriptionally, we knocked down p65 using siRNA. RT-qPCR analysis revealed that p65 knockdown markedly reversed the downregulation of CDKN1C expression (Fig. 5J), and ChIP-qPCR confirmed direct p65 binding to the CDKN1C promoter, which was enhanced by *F. nucleatum* stimulation (Figs. 5K and 5L), indicating that p65 directly binds the CDKN1C promoter to repress its transcription. Finally, the GSIS experiment demonstrated that blocking the activation of the NF-κB pathway could significantly restore the reduction in insulin secretion caused by *F. nucleatum* (Fig. 5M). These results indicate that *F. nucleatum* activates the NF-κB pathway to enhance p65-mediated transcriptional repression of CDKN1C, promoting β-cell identity loss and dysfunction.

## Discussion

Studies have demonstrated that periodontitis negatively affects glycemic control in individuals with T2D [43–45]. The periodontal pathogen *P. gingivalis* has been shown to worsen insulin resistance by modulating adaptive immune responses and downregulating insulin receptor expression, thereby contributing to T2D progression [16, 17]. However, the role of *F. nucleatum*, another key periodontal pathogen, in diabetes pathogenesis has remained largely unexplored. This study initially demonstrates that *F. nucleatum* may play a significant role in accelerating T2D progression.

T2D is characterized by insulin resistance and β-cell dysfunction [46]. The reduction in functional β-cell mass is not solely attributable to apoptosis but may also involve loss or transformation of β-cell identity [1, 3]. In this study, we confirmed a significant decrease in β-cell proportion in T2D, and at the transcriptomic level, we observed marked downregulation of β-cell-specific transcription factors, along with significant upregulation of α-cell markers and genes normally suppressed in mature β-cells. Further Slingshot trajectory analysis clearly revealed a shift toward α-cell transdifferentiation rather than δ-cell fate conversion in the T2D group. These findings align with previous studies showing that β-cells exhibit plasticity under metabolic stress [47–49]. Notably, in the Pre-T2D group, reduced β-cell proportions and increased proportions of other cell types were detected, indicating that β-cell identity loss may occur early in T2D development, rather than representing a terminal pathological event. Pathway enrichment and cell-cell communication analyses further uncovered extensive remodeling of signaling networks and the islet microenvironment during T2D pathogenesis. As a central mediator of inflammation, the NF-κB pathway not only regulates the expression of pro-inflammatory cytokines but also interferes with the transcription of key β-cell functional genes, thereby promoting β-cell dysfunction and identity instability [50, 51]. Communication signals between β-cells and pancreatic stellate cells, endothelial cells, and immune cells were enhanced, forming a pathological cellular network that accelerates disease progression. Activation of stellate cells drives extracellular matrix deposition and fibrosis, while heightened immune cell activity further intensifies islet inflammation, collectively impairing the functional milieu of β-cells and accelerating identity loss [52]. Abnormal overexpression of the pro-inflammatory signal SPP1 in β-cells and disruption of the WNT signaling pathway, which is crucial for islet homeostasis and function maintenance, were observed. SPP1 is a potent pro-inflammatory mediator that activates the NF-κB signaling pathway [53] and serves as a key biomarker of pancreatic tissue injury and impaired repair mechanisms under stress [54]. These alterations contribute to the deterioration of islet architecture and compromise the capacity to maintain β-cell homeostasis, ultimately undermining the stability of the mature β-cell phenotype.

Growing evidence supports the involvement of oral microorganisms in metabolic diseases such as T2D [55]. Recent studies have also detected periodontal pathogens, including *F. nucleatum*, within atherosclerotic plaques, providing further evidence for the systemic dissemination of oral bacteria [56, 57]. This study provides direct molecular evidence that *F. nucleatum* is associated with aberrant expression of functional genes in β-cells. In vitro experiments revealed that *F. nucleatum* infection in MIN6 cells significantly impaired insulin secretion, downregulated key β-cell identity factors, and upregulated α-cell and endocrine progenitor markers, suggesting that *F. nucleatum* may directly participate in promoting β-cell identity transition. Furthermore, comparative analysis of the effects of live *F. nucleatum*, HK-*Fn*, and LPS on β-cell function reveals that HK-*Fn* does not have an effect, and that LPS—a key virulence factor of Gram-negative bacteria—has a less pronounced impact than live *F. nucleatum*. These indicate that the influence of *F. nucleatum* on β-cells depends on active infection or bacterial metabolic activity, and involves pathogenic mechanisms beyond LPS. This underscores the distinct pathogenicity of *F. nucleatum* as a specific pathogenic bacterium, warranting further investigation.

We identified CDKN1C (p57) as a key mediator through which *F. nucleatum* disrupts β-cell identity. CDKN1C, a cyclin-dependent kinase inhibitor, regulates cell cycle progression and plays a crucial role in maintaining the identity of terminally differentiated cells [58]. This study demonstrated that in T2D samples, CDKN1C expression decreased as β-cell identity loss progressed and was positively correlated with mature β-cell marker genes, while negatively associated with markers of identity loss. This pattern is consistent with the well-established role of p57 in promoting cell cycle exit and maintaining differentiation homeostasis, as demonstrated in studies of neural stem cells and hepatocytes [59, 60]. Notably, we demonstrated that the periodontal pathogen *F. nucleatum* disrupts β-cell identity through modulation of CDKN1C expression, thereby providing novel biological evidence linking microbial infection to islet cell identity loss. While previous research on *F. nucleatum* has largely focused on its pro-inflammatory and pro-proliferative effects in cancer and inflammatory diseases, its influence on endocrine cell differentiation remains largely unexplored [61]. Here, we propose a new mechanism: *F. nucleatum* can activate the NF-κB pathway, enhancing the transcriptional repression of CDKN1C by p65, which leads to downregulation of CDKN1C expression. This process ultimately promotes the loss of mature identity and dysfunction in β-cells. Collectively, our results not only reinforce the critical role of CDKN1C in β-cell identity maintenance but also establish a direct association between periodontal microbial infection and β-cell identity loss, offering new mechanistic insights into T2D pathogenesis and potential avenues for therapeutic intervention.

This study provides important mechanistic insights, but several limitations should be noted. First, the inferred lineage trajectories and pseudotime dynamics are computational estimates and require functional lineage tracing or perturbation experiments for validation. Second, the lack of an independent validation cohort and comprehensive clinical metadata limits the generalizability of our findings, and unmeasured confounders may still influence the associations. Finally, further investigation is needed into the mechanisms of *F. nucleatum* enrichment in pancreatic islets, its interaction with β-cells, and the regulatory networks governed by CDKN1C. Larger, more diverse single-cell datasets will enhance the translational significance of our findings.

## Conclusions

In conclusion, this study reveals a novel mechanism by which the periodontal pathogen *F. nucleatum* contributes to the T2D progression, defining the specific transdifferentiation fate of compromised β-cells and identifying the *F. nucleatum*–NF-κB–CDKN1C axis as a critical regulator of this process. Therefore, treatment targeting oral pathogens or the NF-κB–CDKN1C axis to reduce the loss or dysfunction of mature β-cells may hold significant importance for the control of T2D.

## Electronic supplementary material

Below is the link to the electronic supplementary material.

Supplementary Material 1

Supplementary Material 2

Supplementary Material 3

## Data Availability

scRNA-seq and transcriptomic data (GSE221156, GSE164416) of human pancreatic islets were downloaded from the GEO datasets from the National Center for Biotechnology Information (NCBI) database. All data, analytic methods, and study materials will be made available to other researchers upon reasonable request.

## Abbreviations

GSIS: glucose-stimulated insulin secretion
KEGG: Kyoto Encyclopedia of Genes and Genomes
GSEA: Gene set enrichment analysis
GO: Gene Ontology Analysis
BP: Biological Processes
MF: Molecular Functions
CC: Cellular Component
LPS: Lipopolysaccharide
