# Supplementary figures and images for: Single-cell RNA-seq and in vitro study reveal Fusobacterium nucleatum impairs β-cell identity in type 2 diabetes via the NF-κB–CDKN1C axis
Source: J Transl Med. 2026 Mar 9;24:515. doi: 10.1186/s12967-026-07981-x (PMC13085606; doi:10.1186/s12967-026-07981-x)

Figure4


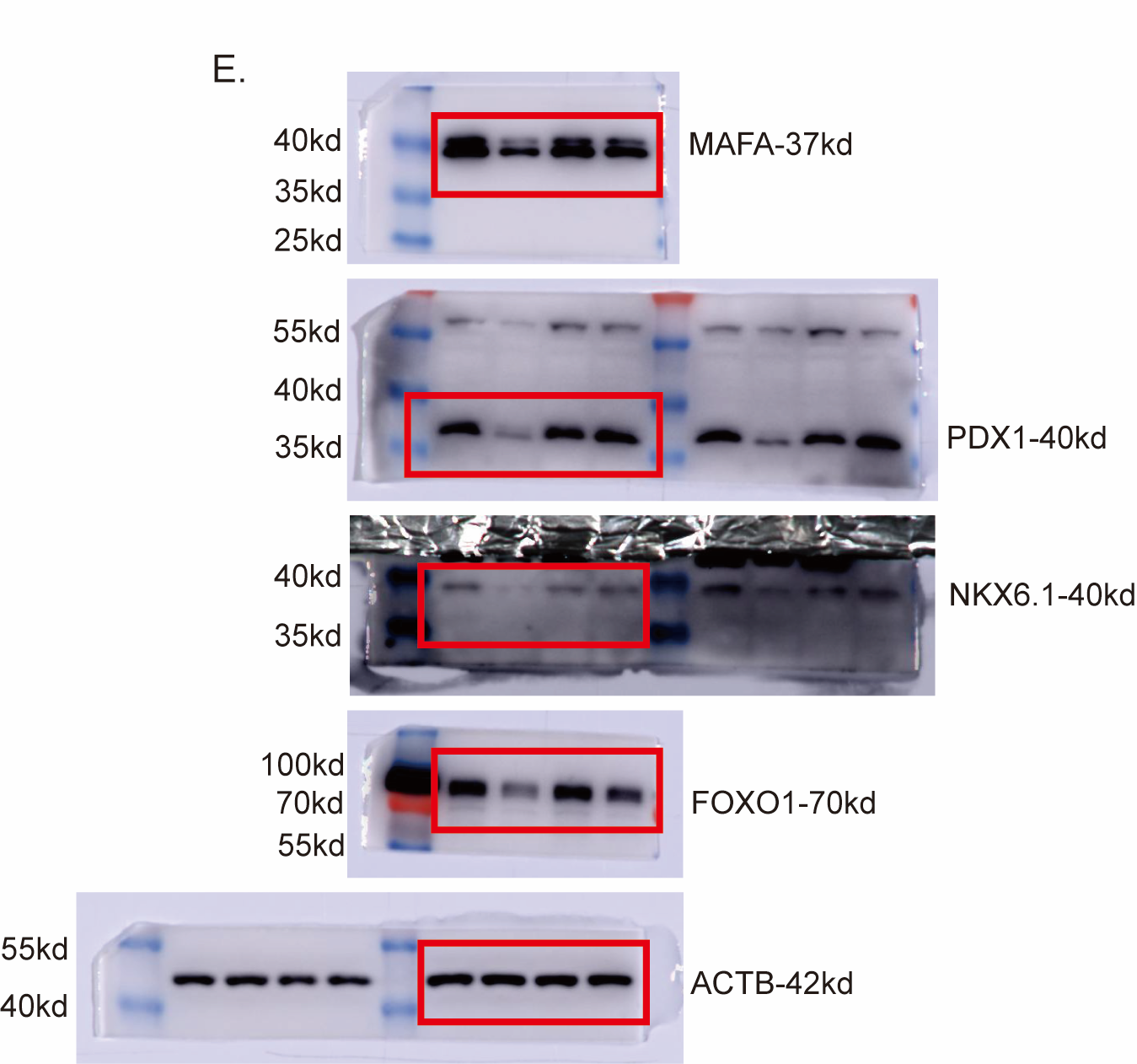


Figure4


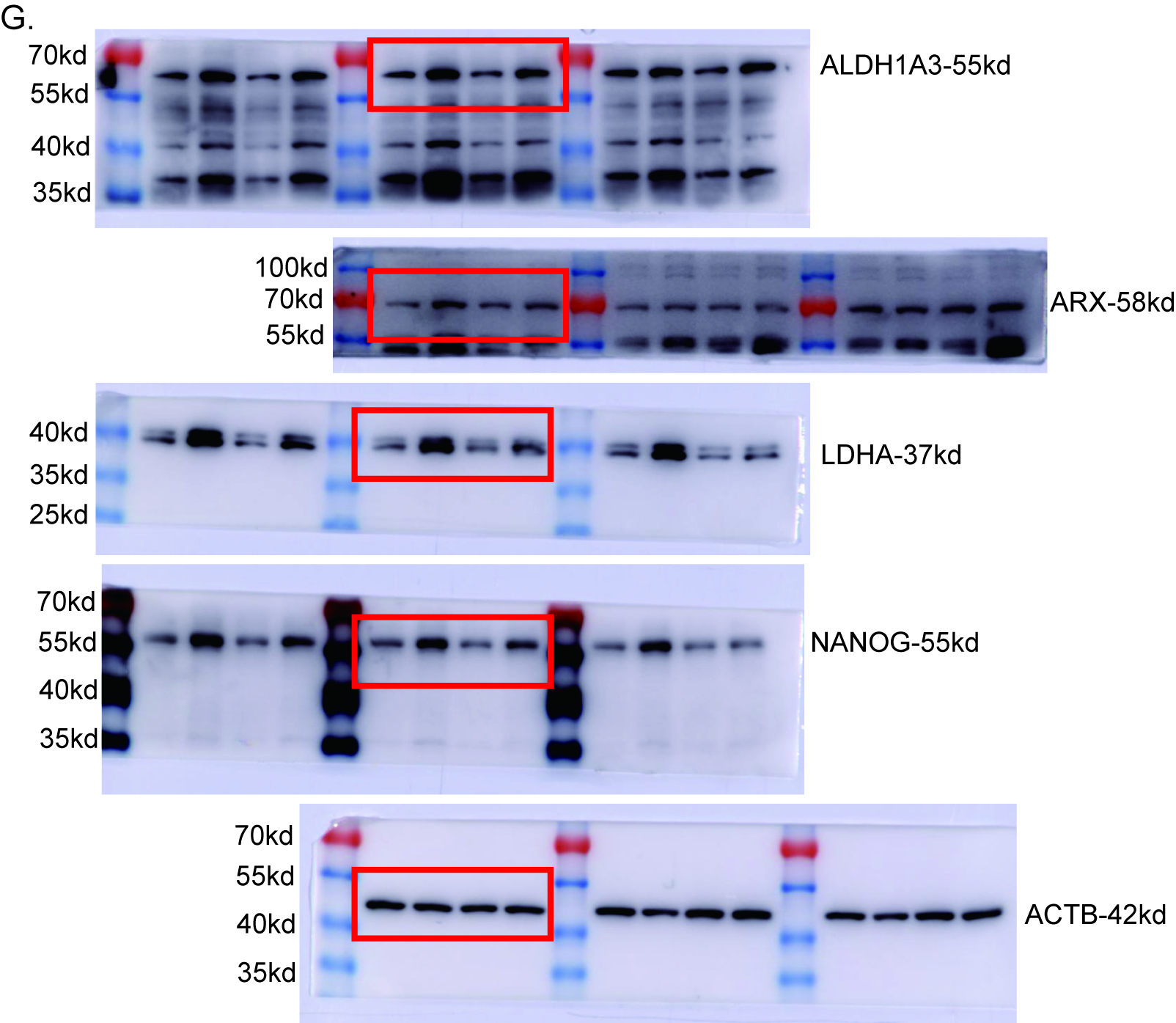


Figure5


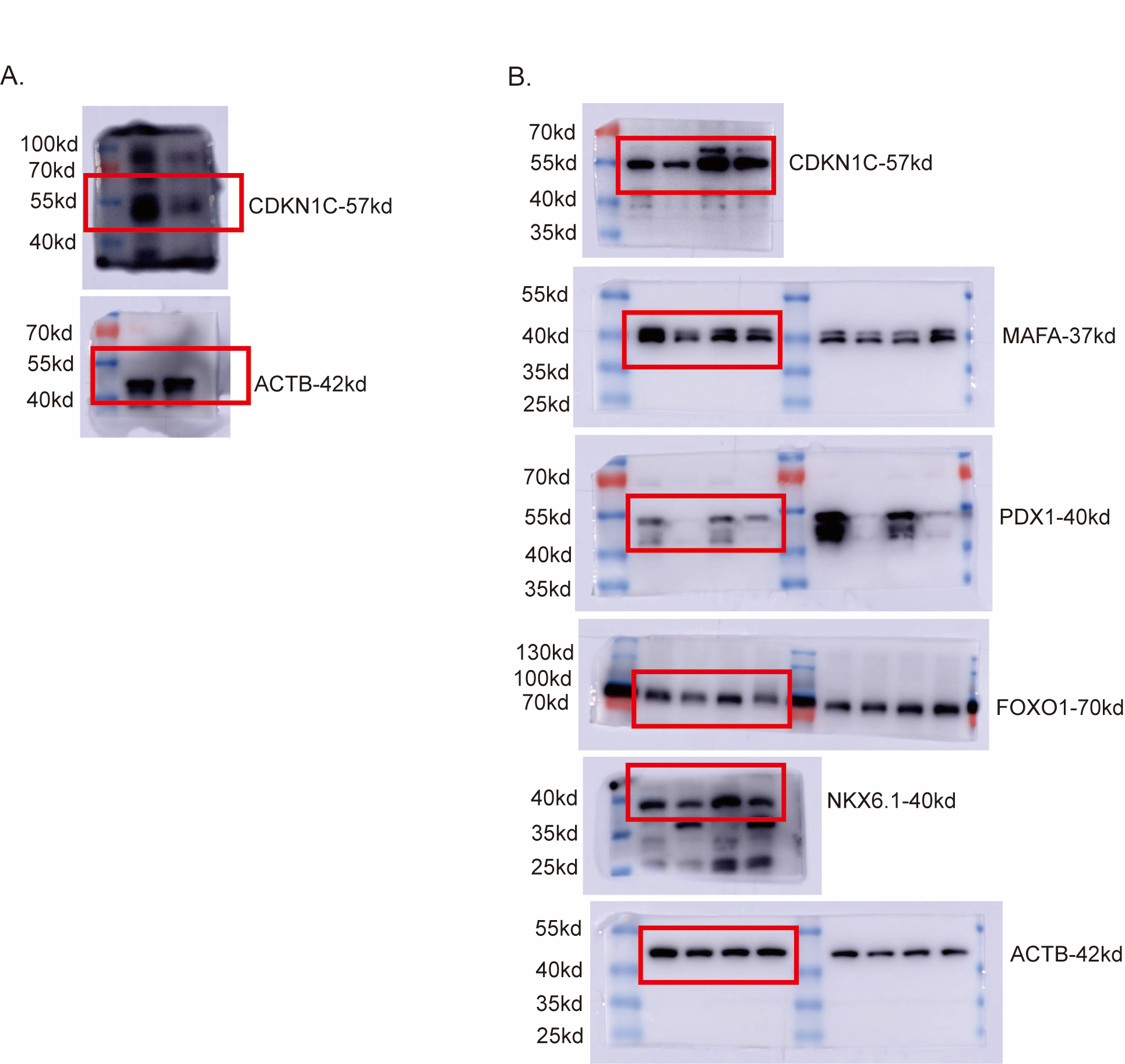


Figure5


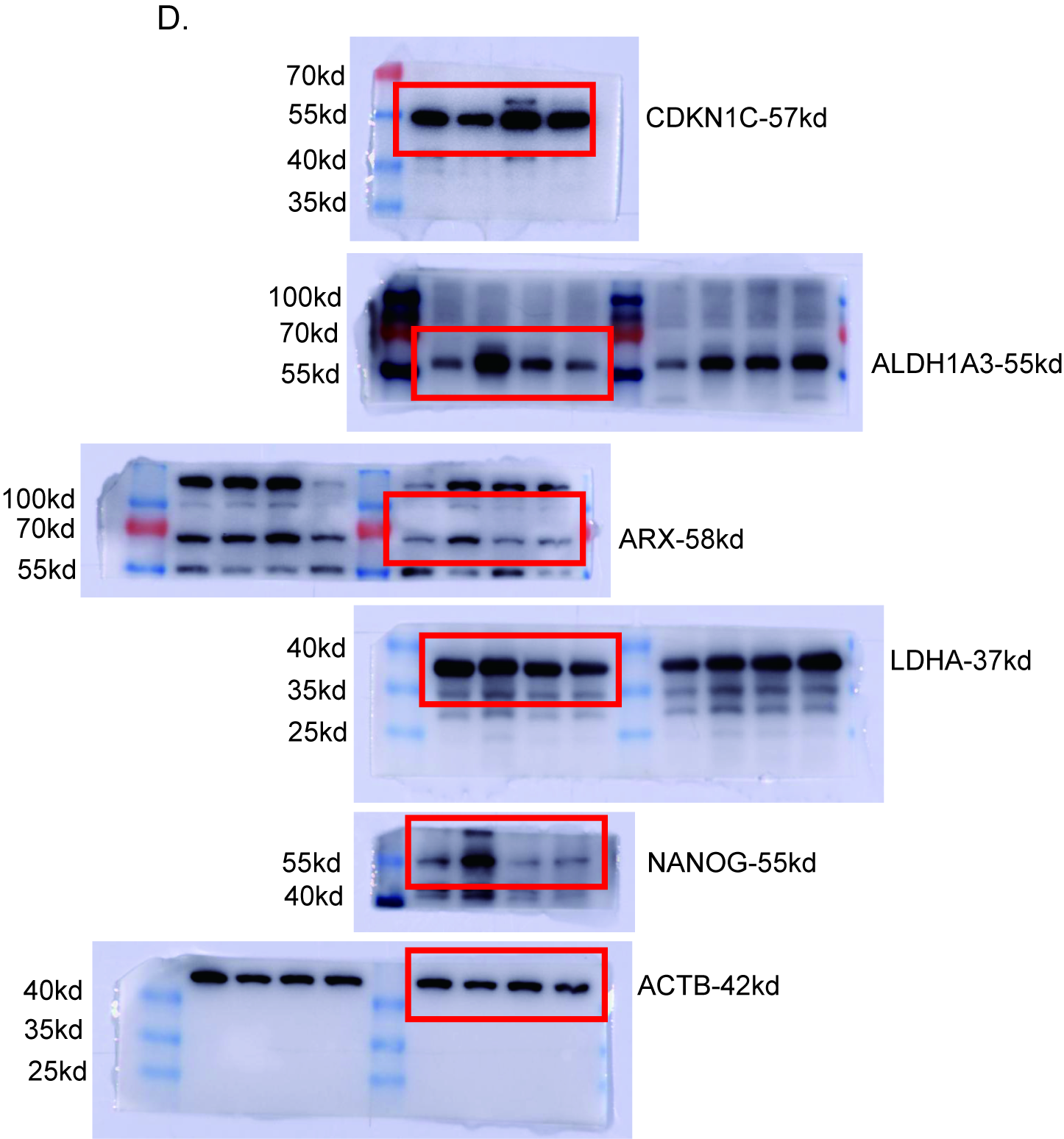


Figure5


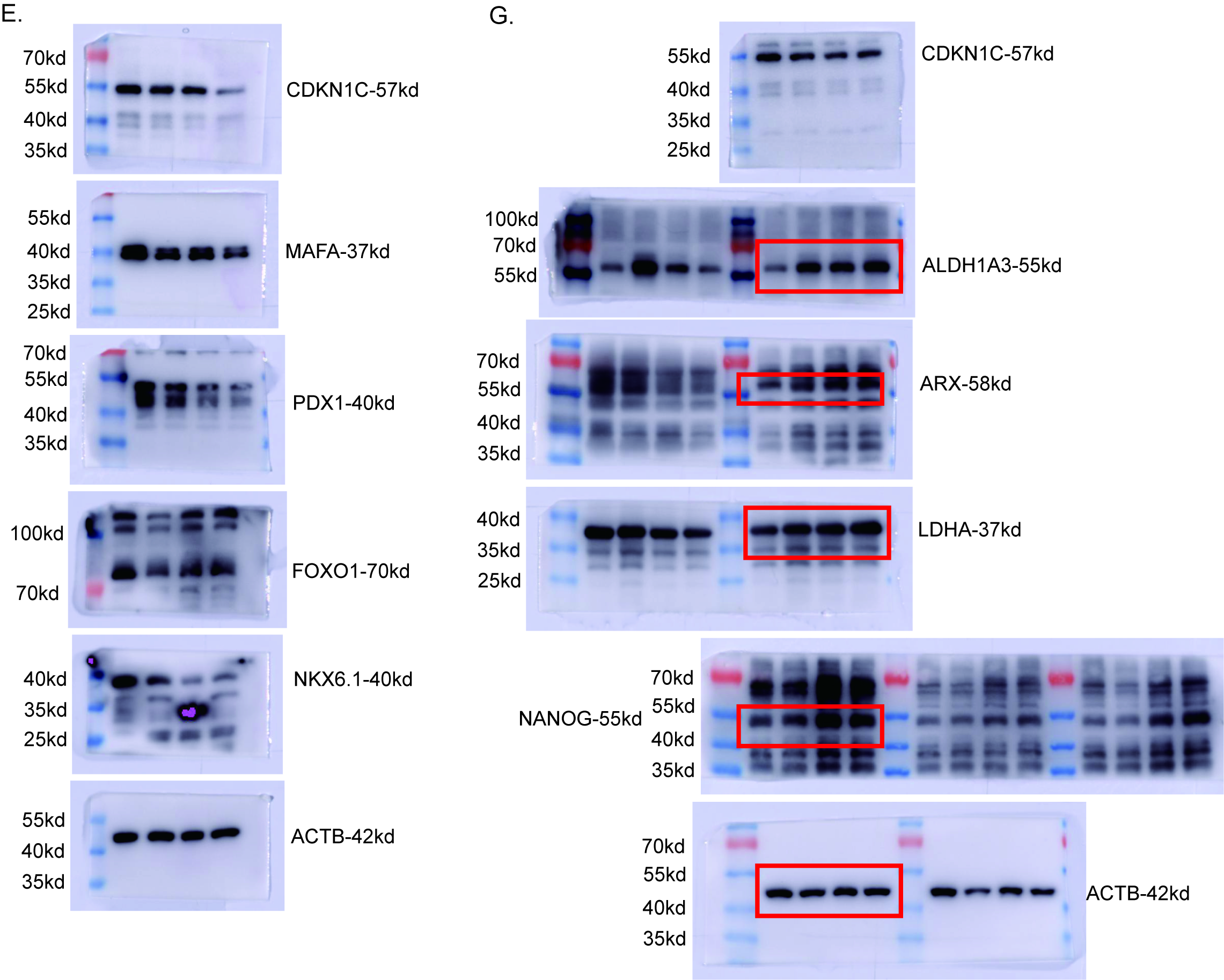


Figure5


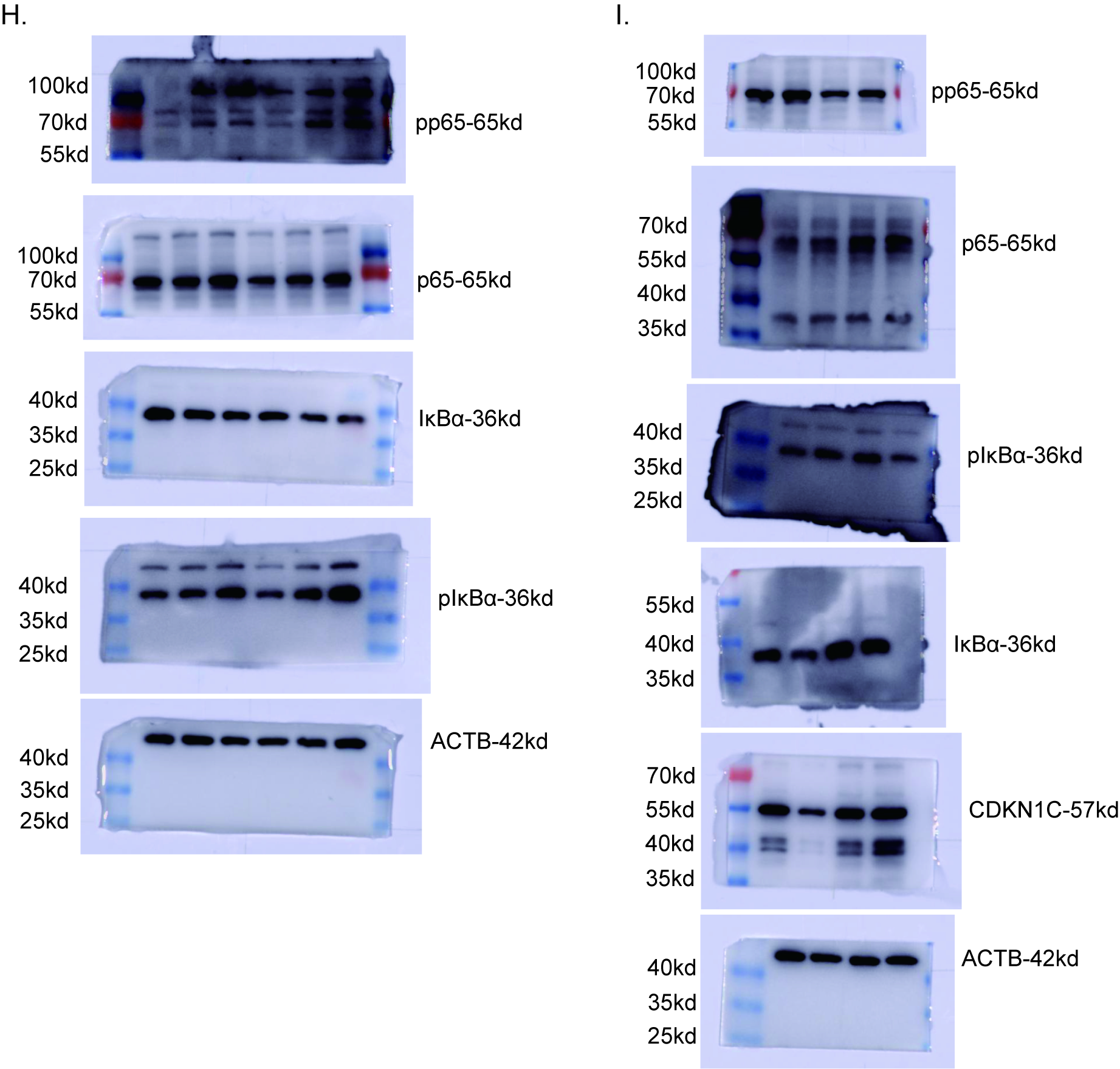


FigureS5


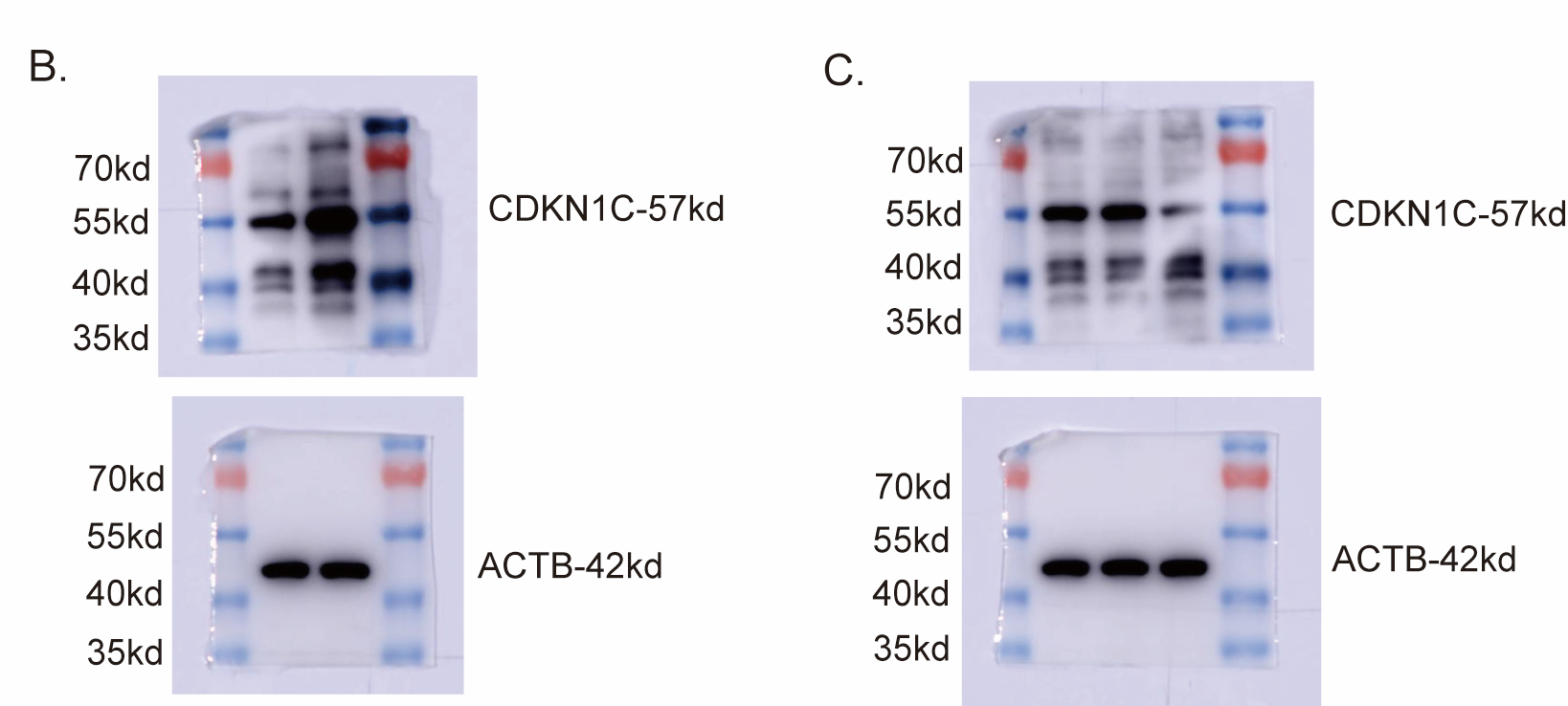

Supplement: Supplementary file 3 — Supplementary Material 3 [file 12967_2026_7981_MOESM3_ESM.docx]
