# Supplementary material for: Single-cell RNA-seq and in vitro study reveal Fusobacterium nucleatum impairs β-cell identity in type 2 diabetes via the NF-κB–CDKN1C axis
Source: J Transl Med. 2026 Mar 9;24:515. doi: 10.1186/s12967-026-07981-x (PMC13085606; doi:10.1186/s12967-026-07981-x)
Supplement: Supplementary file 2 — Supplementary Material 2 [file 12967_2026_7981_MOESM2_ESM.docx]

**Schematic of the CDKN1C Plasmid Construction**

**Fig. S1: Expression heterogeneity of different cells on pancreatic islets in diabetes.** (A) Dotplot showing the relative expression of canonical genes per cell type. (B), (C) Top 150 genes in each cluster and their enriched BP (B) and MF (C) pathways (*q*-value < 0.05). Marked genes are used for the annotation of cell clustering.

**Fig. S2: The cell proportion, AUCell scores, and pseudotime analysis together demonstrate β-cell identity loss.** (A) UMAP of cells depicted in the ND, Pre-T2D, and T2D groups. (B) The composition of different cell clusters across the ND, Pre-T2D, and T2D groups. (C) α-, δ-, PP cells composition across samples in the ND, Pre-T2D, and T2D groups. Red dots indicate the median values. (D) Dotplot showing the expression of β-cells maturation markers across the ND, Pre-T2D, and T2D groups. (E) Dotplot showing the expression of β-cell “disallowed” genes, α-cell markers, and dedifferentiation-related genes in β-cells of the ND, Pre-T2D, and T2D groups. (F) Box plot showing the β-cell identity loss scores according to the genes of β-cells maturation markers in the ND, Pre-T2D, and T2D groups (Wilcoxon test, ***p* ≤ 0.01). (G) Box plot showing the β-cell identity loss scores according to the genes of β-cell identity loss in the ND, Pre-T2D, and T2D groups (Wilcoxon test, ***p* ≤ 0.01 and *****p* ≤ 0.0001). (H) Box plot showing the β-cell identity loss scores of samples in the ND and T2D groups of the GSE164416 dataset according to the genes in (E) (Wilcoxon test, ***p* ≤ 0.01). (I) Box plot showing the β-cell identity loss scores of samples in the ND and T2D groups of the GSE164416 dataset according to the genes in (D) and (E). (J) Mean weight of β-cells in the three trajectories across the ND, Pre-T2D, and T2D groups (Wilcoxon test, *****p* ≤ 0.0001). (K) Pseudotime of high-weight β-cells in the three trajectories at sample level across the ND, Pre-T2D, and T2D groups. Red dots indicate the median values (J, K).

**Fig. S3: The GSEA and cell communication results of the ND and T2D groups.**

(A), (B), (C) The GSEA result showing the activation of TNF, NF-κB, and PI3K-Akt signaling pathway. (D) Clustering of signaling pathways from ND and T2D groups by functional similarity. (E) Signaling networks with substantial (or minimal) differences are identified based on the Euclidean distance between them in a shared two-dimensional space. A greater distance indicates a larger disparity in the functional similarity of the communication networks between the ND and T2D groups. (F) The heatmap shows the outgoing signaling patterns of different cell types in the ND and T2D groups. (G) The differences in ligand receptor levels between β-cells and other cells in the T2D group. (H), (I) The expression of SPP1 (H) and WNT4 (I) genes in β-cells of the ND and T2D groups. Red dots indicate the median values.

**Fig. S4: Validation of *F. nucleatum* annotation and its relationship with β-cell identity loss.** (A) The pie chart shows the proportion of *F. nucleatum* annotations for the samples in the GSE164416 dataset. (B) Regression analysis results of the abundance of *F. nucleatum* and the β-cell identity loss score that is calculated according to the genes of Figure S2E (*p* = 0.019). (C) Regression analysis results of the abundance of *F. nucleatum* and the β-cell identity loss score that is calculated according to the genes of Figure S2D and S2E (*p* = 0.0049). (D) Regression analysis results of the β-cell identity loss score and the expression of CDKN1C (*p* = 0.27).

**Fig. S5: Validation of the number of viable** ***F. nucleatum* and CDKN1C knockdown and overexpression.** (A) Quantification of viable *F. nucleatum* cells at an MOI of 50 by CFU assay. (B) CFU assay quantifying viable *F. nucleatum* in MIN6 co-culture, expressed as percentage of CFU relative to 0 h. (C) RT‑qPCR analysis of β-cell function gene expression in MIN6 cells after co-culture with *F. nucleatum* for different durations. (D) GSIS assay showing insulin secretion by MIN6 cells during co-culture with *F. nucleatum*. (E) CCK‑8 assay measuring MIN6 cell viability following *F. nucleatum* co-culture. (F) Western blot analysis validating the efficiency of CDKN1C overexpression plasmids. (G) Western blot analysis of the validation of CDKN1C knockdown efficiency by two specific siRNAs. Data were shown as mean ± SD. Student’s t-test (F) and One-way ANOVA (D, E and G) were used to examine the statistical significance between groups, **p* < 0.05, ***p* < 0.01, and ****p* < 0.001.
